# Defoliation of interior Douglas-fir elicits carbon transfer and stress signalling to ponderosa pine neighbors through ectomycorrhizal networks

**DOI:** 10.1038/srep08495

**Published:** 2015-02-16

**Authors:** Yuan Yuan Song, Suzanne W. Simard, Allan Carroll, William W. Mohn, Ren Sen Zeng

**Affiliations:** 1 College of Life Sciences, Fujian Agriculture and Forestry University, Jinshan, Fuzhou 350002, P.R. China; 2 Department of Forest and Conservation Sciences, University of British Columbia, Vancouver, British Columbia, V6T 1Z4, Canada; 3 Department of Microbiology & Immunology, Life Sciences Institute, University of British Columbia, Vancouver, BC, V6T 1Z3, Canada

## Abstract

Extensive regions of interior Douglas-fir (*Pseudotsuga menziesii* var. glauca, IDF) forests in North America are being damaged by drought and western spruce budworm (*Choristoneura occidentalis*). This damage is resulting from warmer and drier summers associated with climate change. To test whether defoliated IDF can directly transfer resources to ponderosa pine (*Pinus ponderosae*) regenerating nearby, thus aiding in forest recovery, we examined photosynthetic carbon transfer and defense enzyme response. We grew pairs of ectomycorrhizal IDF ‘donor’ and ponderosa pine ‘receiver’ seedlings in pots and isolated transfer pathways by comparing 35 μm, 0.5 μm and no mesh treatments; we then stressed IDF donors either through manual defoliation or infestation by the budworm. We found that manual defoliation of IDF donors led to transfer of photosynthetic carbon to neighboring receivers through mycorrhizal networks, but not through soil or root pathways. Both manual and insect defoliation of donors led to increased activity of peroxidase, polyphenol oxidase and superoxide dismutase in the ponderosa pine receivers, via a mechanism primarily dependent on the mycorrhizal network. These findings indicate that IDF can transfer resources and stress signals to interspecific neighbors, suggesting ectomycorrhizal networks can serve as agents of interspecific communication facilitating recovery and succession of forests after disturbance.

Plants have evolved the ability to communicate with neighboring plants for alleviating stresses within communities by transmitting volatile compounds aboveground or a variety of organic and inorganic compounds belowground. Mycorrhizal networks, comprised of mycorrhizal fungi connecting the roots of multiple plants, are potentially direct pathways for belowground transmittance of these biochemical messages between plants^1^. There is increasing evidence that mycorrhizal networks can transmit, for example, herbivore- or pathogen-induced defense signaling compounds to warn neighbors of pest infestations^2^,^3^,^4^, kin recognition signaling compounds involving micronutrients to communicate genetic relationships of neighbors^5^,^6^, toxins such as allelochemicals to convey negative interactions to competing neighbors^7^, and essential resources such as carbon, nitrogen, phosphorus or water for altering physiology, survival or growth of conspecific or heterospecific neighbors^8^. Mycorrhizal networks have also been shown to rapidly transmit phosphorus and nitrogen from dying plants to healthy conspecific neighbors^9^, providing a conduit for legacy transference across generations. Similarly, clipping has prompted transport of labile carbon from stressed to healthy heterospecific neighbours through arbuscular mycorrhizal networks^10^. The neighbors receiving these messages could potentially then modify their behavior through altered morphology, physiology or biochemistry, thus reducing their stress and improving fitness. Although there is increasing evidence for interplant communication through mycorrhizal networks, the majority of studies conducted so far have focused on herbaceous or grass species forming arbuscular mycorrhizal networks. Belowground communication between trees linked by ectomycorrhizal networks in forests, however, has received little attention.

Forests world-wide are experiencing increasing stress and tree mortality as climate changes^11^,^12^. Climate change is disrupting co-evolved host-pest interactions by altering life cycles of forest trees, insects, and fungal pathogens, causing well-documented outbreaks of bark beetles, blights and rusts in pine (*Pinus*) and spruce (*Picea*) species in North America^13^,^14^,^15^,^16^. Extensive regions of interior Douglas-fir (*Pseudotsuga menziesii* (Mirb.) Franco var glauca (Mayr)) forests are also being defoliated by drought, western spruce budworm (*Choristoneura occidentalis*) and Douglas-fir tussock moth (*Orgyia pseudotsugata*) in direct response to warmer summer temperatures^15^. Sustained severe defoliation causes tree mortality, creating new growing space for potential migration of tree species from warmer locations and helping facilitate predicted forest vegetation shifts^17^. These forest vegetation shifts are further facilitated by extensive salvage logging of dying and dead trees^13^. In interior British Columbia, mortality and salvage logging of the dry interior Douglas-fir forests resulting from climate change should create favorable conditions for upward and northward migration of ponderosa pine (*Pinus ponderosa* Douglas ex C.Lawson), as predicted by the climate envelope models of Wang et al.^17^.

Douglas-fir and ponderosa pine are host to hundreds of ectomycorrhizal fungal species in North and South America, including many ‘generalist’ fungi common to both tree species^18^, and thus they can become linked in a mycorrhizal network where the two tree species co-occur in nature^19^,^20^. In a replacement series experiment, Douglas-fir and ponderosa pine yielded greater biomass in co-culture than monoculture when co-colonized by *Laccaria laccata* (Scop. ex Fr.) Bk. & Br., where enhanced foliar nitrogen and phosphorus nutrition may have occurred because of interplant nitrogen or phosphorus transfer through the mycorrhizal network^19^. These results suggest that mycorrhizal-mediated interplant transfer of nutrients has the potential to influence resource distribution and plant performance within communities^21^,^22^,^23^. This discovery also opens the possibility that mycorrhizal networks can serve as a conduit for the transfer of nutrient legacies or stress signals from interior Douglas-fir to healthy ponderosa pine neighbors in response to climate-induced defoliator outbreaks.

Trees have coevolved with native insects and pathogens so that they can respond to infection by producing an array of defense compounds that mediate their interactions with the invader^24^. Trees have also coevolved with ectomycorrhizal fungi that are responsible for nutrient and water uptake in exchange for carbon^20^. Previous research with arbuscular mycorrhizal tomatoes also shows that, when plants are connected by a mycorrhizal network, stress signals can transfer from infected plants to conspecific neighbors through this network, thus increasing activities of defense compounds, inducing defense-related genes, activating the jasmonate pathway, and increasing pest resistance in receiver plants^2^,^3^. Additionally, in other previous research, we have shown that interior Douglas-fir can transfer carbon, nitrogen and water through ectomycorrhizal networks to conspecific or heterospecific neighbors, and that this has been associated with increased survival, growth and foliar nutrition of recipient neighbors^23^,^25^,^26^,^27^. Taken together, these studies suggest that signal and resource transfer from interior Douglas-fir to ponderosa pine through ectomycorrhizal networks could play a role in facilitating and shaping the predicted forest vegetation shifts in this region as climate changes.

The objective of this study was to determine whether injury to interior Douglas-fir by insect or manual defoliation would induce interspecific transfer of carbon and stress signals to neighboring healthy ponderosa pine seedlings through a mycorrhizal network. Our first hypothesis was that manual and insect defoliation would cause interior Douglas-fir to export labile carbon directly to neighboring ponderosa pine through mycorrhizal networks. We expected increasing levels of carbon export with increasing degree of injury. We also expected the presence of root competition to reduce amount of transfer. Our second hypothesis was that manual and insect defoliation would cause interior Douglas-fir to communicate via organic stress signals with ponderosa pine to increase its defense response. We expected greater defense response with insect than manual defoliation because of coevolution between tree and insect species. We also expected greater stress signal transfer directly through mycorrhizal networks than indirectly through soil pathways.

## Methods

### Seed and soil

Interior Douglas-fir seed was acquired from the Surrey Seed Centre of the B.C. Ministry of Forests, Lands, and Natural Resource Operations (Surrey, B.C.). Seeds were surface-sterilized with 10% H_2_O_2_ and rinsed with sterile distilled water before sowing. Seeds were sown in a 3:1 mixture of autoclaved potting soil and non-sterile forest soil (Dystric Brunisol, sandy loam texture, moder humus form; Soil Classification Working Group, 1998) collected from a mono-specific stand of interior Douglas-fir (120.58°W, 49.43°N, IDFdk biogeoclimatic subzone^28^) to facilitate ectomycorrhizal colonization. Soil was collected by removing the litter layer then extracting the fermentation layer, humus layer, and mineral soil, to a depth of 10 cm.

### Experimental design

A 3 × 3 factorial set of treatments, with three belowground pathways (soil only, mycorrhizal network, mycorrhizal network plus roots) and three defoliation treatments (healthy, manual defoliation, insect defoliation), was replicated ten times in a completely randomized design (total of 90 experimental units). Each experimental unit consisted of a plant pair established in a 3.8 L pot and grown for 4 months from November 2011–February 2012. Pairs consisted of one ‘donor’ interior Douglas-fir seedling and one ‘recipient’ ponderosa pine seedling grown in approx. 800 g soil (dry weight). For the belowground pathway treatments, the ponderosa pine receivers were grown either in an 8 × 18 cm nylon mesh bag (Plastok® Meshes and Filtration Ltd., Birkenhead) containing 400 g soil (dry weight) inside the same pot; or in no mesh, that is, directly into soil beside the donor in the same pot. Recipient bags were made from one of two mesh sizes, 0.5 μm and 35 μm; both blocked root passage while permitting diffusion of solutes, whereas the smaller mesh also blocked hyphal passage of ectomycorrhizal fungi^29^. Receiver seedlings planted directly into soil (no mesh) could form mycorrhizal networks and their roots were free to intermingle with donor roots. For the defoliation treatments, the donor interior Douglas-fir seedlings were either (i) healthy (left undefoliated), (ii) manually defoliated, or (iii) defoliated by inoculation with western spruce budworm. Defoliation occurred 24 h prior to isotopic labeling. For manual defoliation, all needles were clipped at the petiole using an exacto-knife. For the insect defoliation, two budworms were applied per seedling. The 3^rd^ instars of western spruce budworm were kindly provided by John Dedes at the Great Lakes Forestry Centre, Forestry Canada in Sault Ste. Marie, Canada. During and after defoliation, the ‘donor’ plant was covered with an air-tight plastic bag.

Neither fertilizer nor supplementary light were provided to the plant pairs. Pots were watered to field capacity once per week following an early germination period of light daily watering. A fine gravel layer was applied to soil surfaces to discourage ‘damping off’ fungi. Soil water content was measured to 10 cm depth inside and outside the mesh bags immediately prior to defoliation treatment application using a hand-held time-domain reflectometry probe (Hydrosense CS620, Campbell Scientific).

### ^13^CO_2_ isotope labelling

Four months after establishing the experiment, donor plants were pulse-chase labeled with ^13^C-labelled CO_2_ (99% ^13^C; Cambridge Isotope Laboratories Inc., Andover). Immediately prior to labelling, donor seedlings were sealed inside a plastic Foodsaver® vacuum bag (6 L capacity), fitted with an injection valve, using Tuck® Contractors Sheathing Tape and inflated with ambient air. Labelled seedlings were segregated from non-labelled controls by a 4-m buffer, and received one injection of 50 ml ^13^CO_2_ over a 2-h pulse period. An additional seedling was used to monitor labelling bag CO_2_ concentration using a portable infrared gas analyser (Qubit Systems, Kingston). After the pulse, coinciding with CO_2_ concentration dropping below 300 ppm, labelling bags were removed. Donor, receiver and monitor seedlings were harvested after a 6-day chase period.

### Seedling sampling and elemental analysis

Aboveground (shoot) and belowground (root) biomass of donor, recipient and monitor seedlings were divided by cutting the stem at the soil surface. All fine root tips were sampled from each donor and recipient seedling and morphotyped based on EMF structures^30^. Samples for elemental analysis were kept on dry ice before storage at −20°C. Total carbon and nitrogen content and carbon isotopic composition were measured with combustion analysis using an elemental analyzer (Elementar, Hanau) in C, N mode, interfaced with an isotope-ratio mass spectrometer (IRMS; Isoprime, Cheadle). Samples were considered enriched if their *δ* ^13^C value was greater than the upper 99% confidence interval of the control mean. Atom % ^13^C excess was calculated for each partition as per Deslippe and Simard^31^. Teste et al.'s^23^ modification of Boutton's^32^ isotopic calculations was applied to convert *δ* ^13^C into mg of “excess ^12^C-equivalent” in each partition (mass added if the label was ^12^C rather than ^13^C). Quantification of foliar macro- and micro-nutrients was performed using microwave digestion/ICP (Inductively Coupled Plasma-Optical Emission Spectrometer) at the B.C. Ministry of Environment Analytical Chemistry Laboratory (Victoria, Canada).

### Enzyme assays

Leaf samples for enzyme analyses were harvested from all ‘receiver’ plants 0, 24, 48 and 72 h after defoliation. Three defence-related enzymes, peroxidase (POD), polyphenol oxidase (PPO), and superoxide dismutases (SOD), were analysed. Leaf samples (0.2 g fresh weight) were ground in liquid nitrogen and homogenized in 2.0 ml ice cold 0.05 M phosphate buffer (pH 7.2 for POD or 7.8 for PPO and SOD) containing 1% (w/v) polyvinylpyrrolidone (PVP). The supernatant after 12,000 × g centrifugation for 15 min at 4°C was used for enzyme assays^2^. Activities of POD, PPO and SOD were spectrophotometrically determined according to Kraus & Fletcher^33^, Zauberman et al.^34^ and McCord & Fridovich^35^, respectively. A review of the importance of POD, PPO and SOD in plant defense is provided by Song et al.^2^.

### Statistical analysis

Statistical analysis was carried out using the SAS 8.0 package for windows (SAS Institute, Cary, North Carolina). All data were analyzed with two-way analysis of variance with significant differences among means identified by Tukey's multiple range test at *P* < 0.05.

## Results

### Seedling performance, EMF colonization and water availability

**Figure 1:**
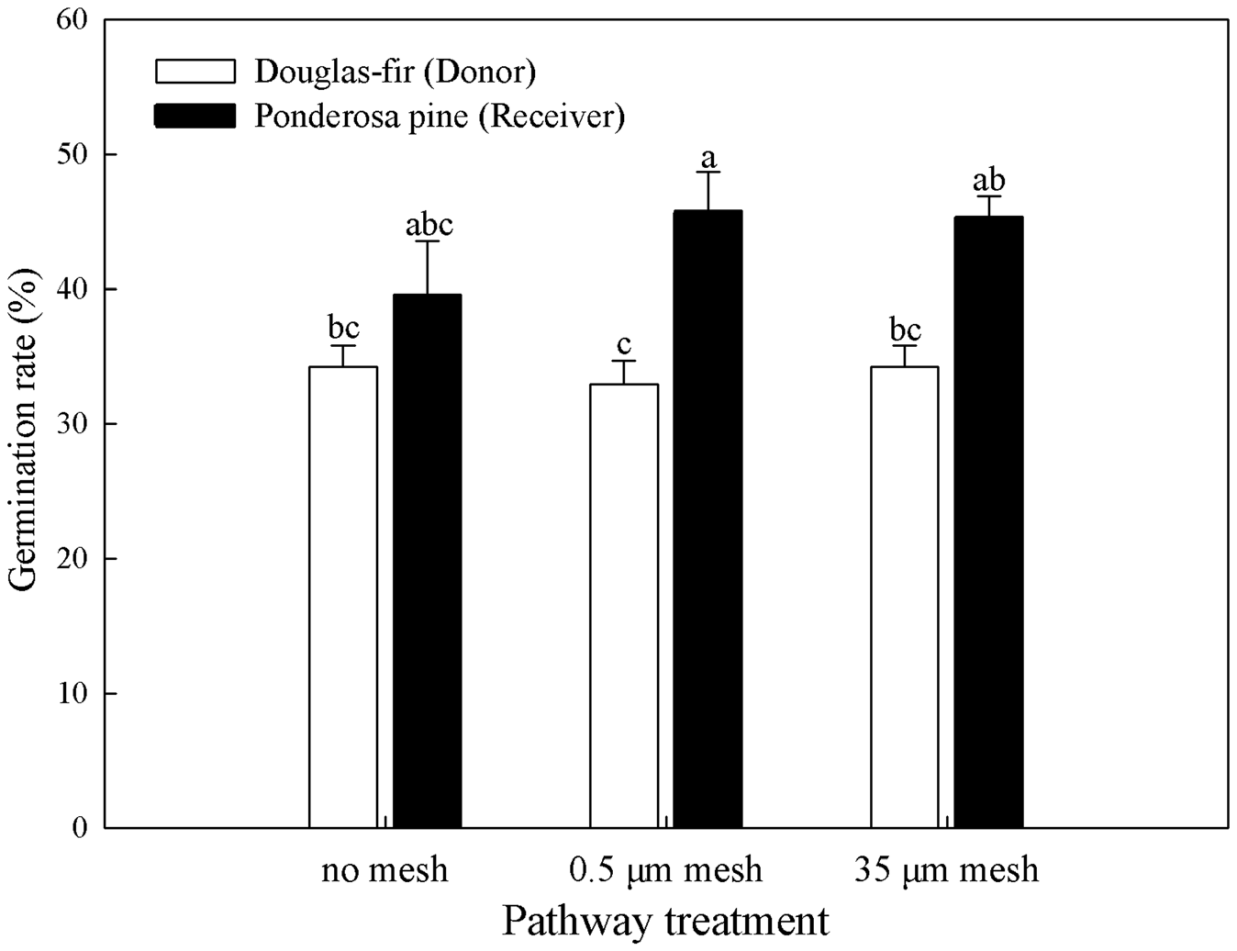
Germination rate (%) of donor interior Douglas-fir and receiver ponderosa pine in the transfer pathway treatments. Means with different letters for each pathway differed significantly at α = 0.05.

**Figure 2:**
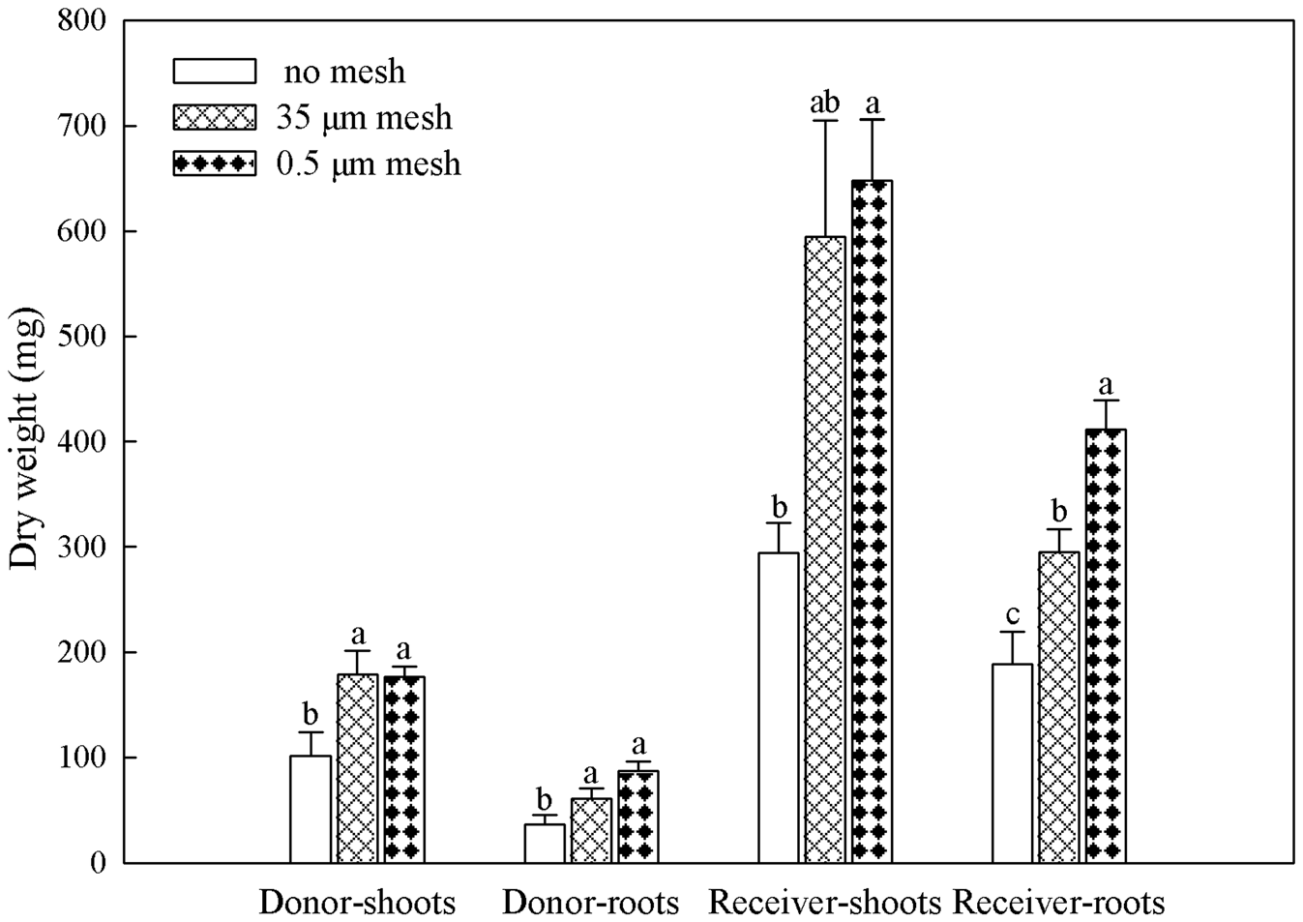
Biomass (mg) of shoots and roots of donor interior Douglas-fir and receiver ponderosa pine prior to application of defoliation treatments. Means with different letters differed significantly within their respective biomass components at α = 0.05.

**Figure 3:**
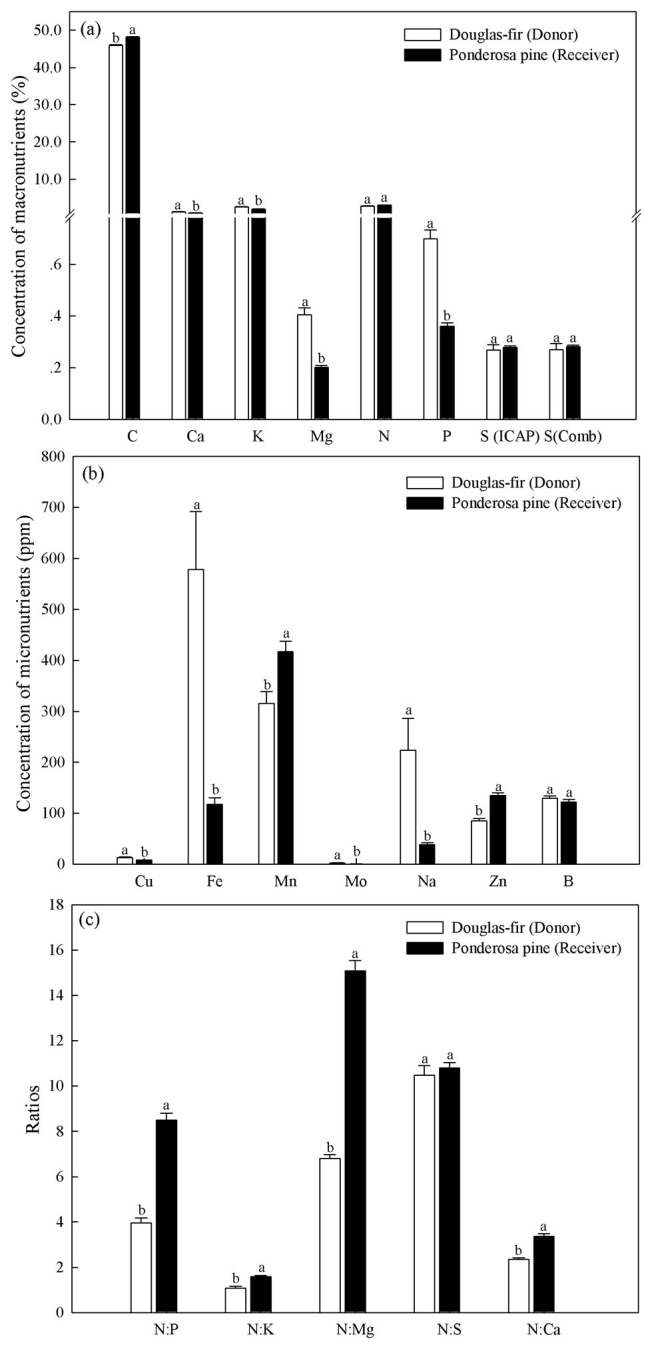
Concentrations (%) of foliar (a) macro-nutrients, (b) micro-nutrients, and (c) ratios for ponderosa pine interior Douglas-fir seedlings prior to labelling. Species means with different letters differed significantly at α = 0.05.

Ponderosa pine receivers germinated at a higher rate than Douglas-fir donors (44% versus 34%, Figure 1). Germination rate of Douglas-fir donors or ponderosa pine receivers did not vary among defoliation treatments, but more ponderosa pine tended to germinate in both mesh bag treatments than the no bag treatment (45% versus 39%, *P* < 0.05, Figure 1). Ponderosa pine receivers were approximately 3 times the size of interior Douglas-fir donors after four months (*P* < 0.05, Figure 2). Neither interior Douglas-fir donor root nor shoot biomass differed among transfer pathway treatments (*P* > 0.05), but receiver ponderosa pine shoots and roots were larger in both mesh bag treatments than the no bag treatment (*P* < 0.05, Figure 2). Ponderosa pine receivers had significantly lower concentrations of P, K, Mg, Ca, Cu, Fe and Mo, and higher Mn, N:P, N:Mg, N:K and N:Ca than interior Douglas-fir donors (*P* < 0.05, Figure 3).

The four month-old interior Douglas-fir and ponderosa pine seedlings were colonized by a single EMF morphotype, *Wilcoxina rehmii*. There were no differences in colonization rates between seedlings or treatments (*P* > 0.05). Water availability to interior Douglas-fir was equivalent to that of ponderosa pine within pathway treatments, indicating mesh bags did not restrict water movement between seedling pairs within a pot (*P* > 0.05, Supplementary Material Figure S1). Water availability was overall 30% higher in the no mesh than the two mesh treatments (*P* < 0.05).

### Carbon transfer

**Figure 4:**
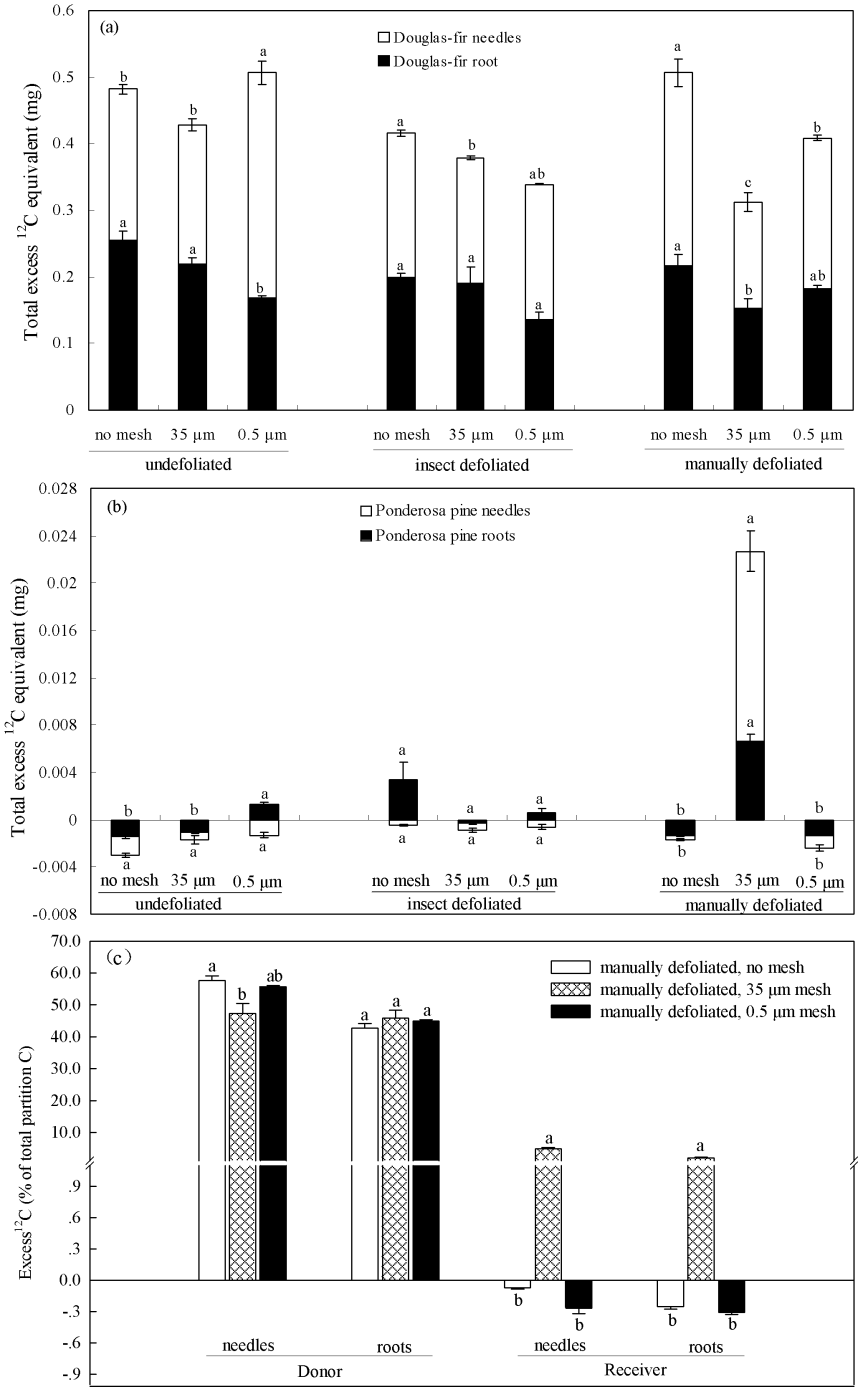
Excess ^12^C-equivalent (mg) of needles and roots for (a) interior Douglas-fir donors and (b) ponderosa pine receivers 6 days following ^13^C-CO_2_ labelling on donors exposed to insect and manual defoliation, and (c) its partition in needles and roots. Pathway means with different letters differed significantly within their respective defoliation treatments at α = 0.05.

**Figure 5:**
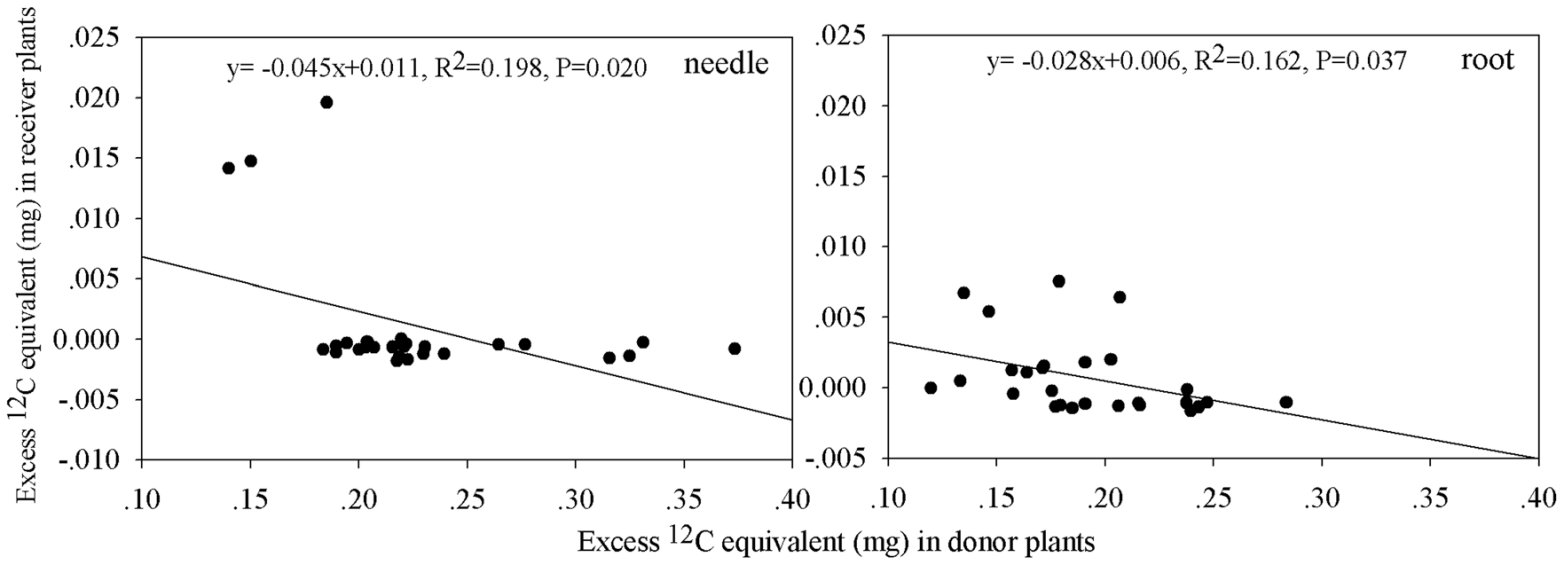
Relationship between donor and receiver ^12^C-equivalent (mg) content for (a) shoots and (b) roots.

To test whether defoliation would cause interior Douglas-fir seedlings to export labile carbon to neighboring ponderosa pine seedlings through mycorrhizal networks, interior Douglas-fir seedlings were labeled with ^13^C-CO_2_ 24 h following manual or insect defoliation. Two-way ANOVA revealed a significant effect of below-ground pathway and defoliation treatment on excess ^12^C equivalent in donors and receivers (*P* < 0.05 for both factors for donors and receivers). Compared with natural abundance levels, all donor needles and roots were successfully labeled, with total excess ^12^C equivalent exceeding 0.3 mg in all treatment combinations (Figure 4a). Donors grown without mesh generally contained more label than those in the other treatments (Figure 4a). Among manually defoliated donors, the mycorrhizal pathway treatment contained less label than the 0.5 μm mesh or no mesh bag treatments. Substantial C transfer occurred only from manually defoliated interior Douglas-fir to healthy ponderosa pine in the 35 μm mesh treatment (*P* < 0.05, Figure 4b). Here, carbon was transferred to both roots and shoots of receiver ponderosa pine. Small but significant C transfer also occurred from healthy interior Douglas-fir to roots of ponderosa pine in the 0.5 μm mesh treatment (*P* < 0.05, Figure 4b). There was no other transfer from healthy or insect defoliated interior Douglas-fir to ponderosa pine, nor in the 0.5 μm mesh or no mesh bag treatments. Regression analysis revealed negative relationships between total ^12^C equivalent in donors and ^12^C equivalent transferred to receiver shoots or roots (*P* < 0.05, Figure 5).

### Carbon partitioning

We examined partitioning of excess ^12^C-equivalent from donor to receiver tissues in the manually defoliated group alone because of the significant C transfer detected (Figure 4c). Of the total mass of ^13^C fixed in the plant pairs, approximately 93.19% (+/−0.55 s.e.m.) was incorporated into donor plant tissue and 6.81% (+/−0.55 s.e.m.) into recipient plant tissue after the 6-day chase period. In donors, equivalent portions of fixed ^13^C occurred in shoots (50.84%) and roots (49.16%). Of the C transferred to recipient seedlings, 29.22% was incorporated into roots and 70.78% into shoots. An estimated 52.62% of total C fixed was translocated belowground, from donor shoots to donor roots plus networked seedlings.

### Defense enzymes

**Figure 6:**
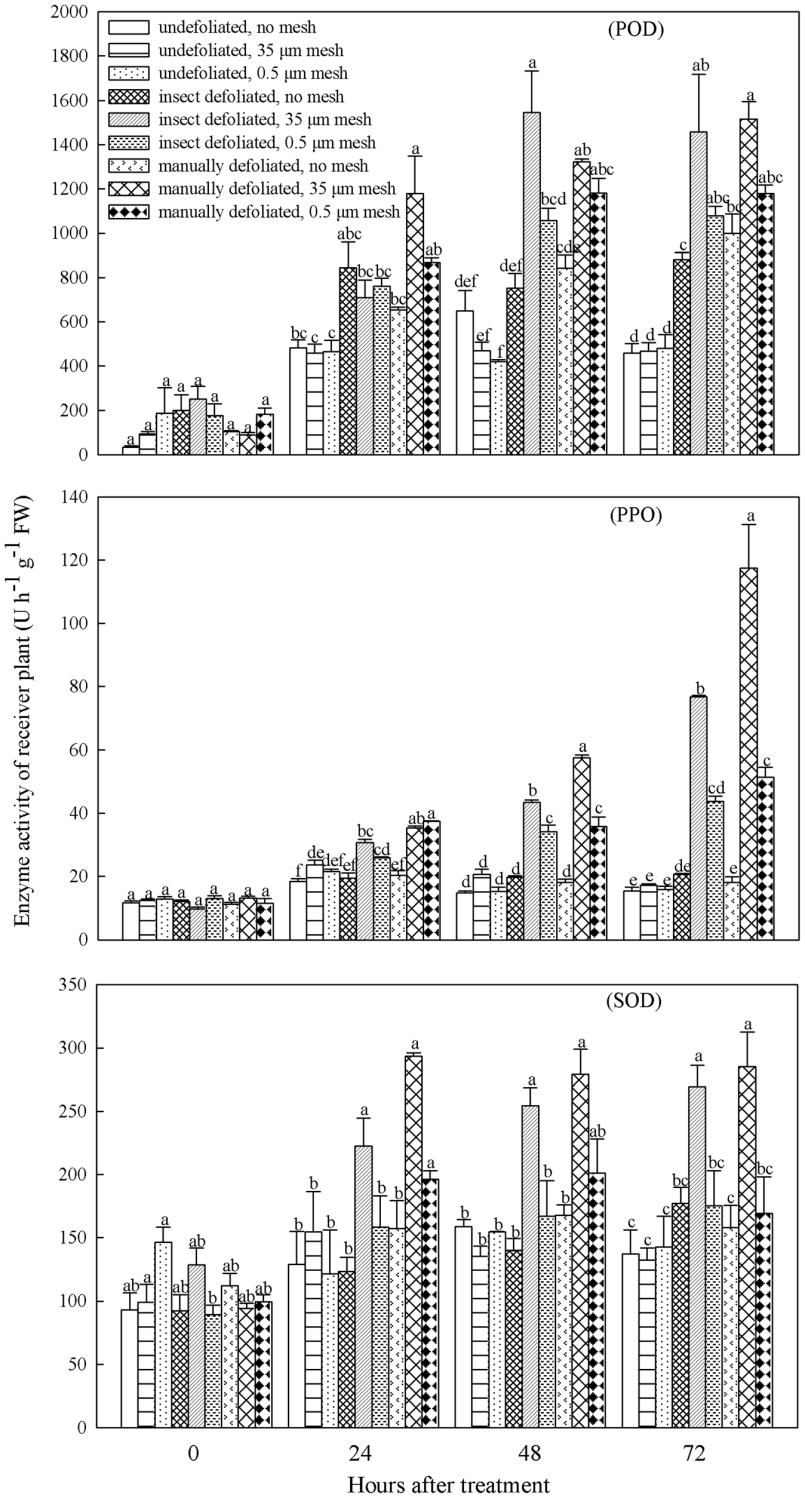
Levels of enzyme activity (POD, PPO, SOD) in needles of receiver ponderosa pine following different defoliation and transfer pathway treatments. Means with different letters for each treatment at the same time point differed significantly at α = 0.05.

Defoliation of donor seedlings caused increased activities of defensive enzymes in recipients, with mycorrhizal networks affecting levels of activity. Compared with the non-defoliated control treatment, the activity of all three enzymes (POD, PPO, SOD) was consistently elevated in ponderosa pine receivers following either manual or insect defoliation of the donors (Figure 6). The defense responses of receivers were substantial, with typically more than 50% of the enzyme activities of their corresponding defoliated donors (Supplementary Material – Figure S2). The resulting enzyme activities were similar following manual and insect defoliation. Generally, in receivers in both defoliation treatments, all three enzyme activities were significantly greater in the 35 μm mesh treatment than either the 0.5 μm mesh or no mesh treatments (Figure 6). Activities in the 0.5 μm mesh treatment generally exceeded those in the no mesh treatment, but these differences were small and often not significant. For each enzyme, the activity of receivers was positively related to that of donors 24, 48 and 72 hours following application of the defoliation treatments (Supplementary Material – Figure S3). The enzyme activity of receivers was also generally positively related to excess ^12^C-equivalent (mg) of donor needles and roots after 48 and 72 hours (Supplementary Material – Figure S4).

## Discussion

In damaged trees, belowground communication with and transfer of carbon legacies to other encroaching tree species may facilitate forest vegetation shifts with climate change. We demonstrate for the first time in ectomycorrhizal conifers that injury to one tree species induces substantial belowground transfer of photosynthetic carbon and elicits a rapid defense response of a different tree species, likely through transfer of stress signals. Moreover, this interspecific communication occurs directly through mycorrhizal networks, bypassing microbial transformations that can occur along soil pathways. The ectomycorrhizal network of the 4-month old interior Douglas-fir and ponderosa pine was comprised of the single taxon, *Wilcoxina rehmii* (Ascomycota, Pezizales order), an E-strain fungal species^36^ well known as an early colonizer of interior Douglas-fir and ponderosa pine seedlings in recently disturbed forest soils^36^,^37^,^38^.

### C transfer and partitioning

Manual but not insect defoliation caused interior Douglas-fir to export labile carbon directly to neighboring ponderosa pine through mycorrhizal networks, partially supporting our first hypothesis. The lack of insect treatment response may be explained by the unexpected minimal levels of defoliation by western spruce budworm compared to manual excision, as evidenced by donor isotope contents in the 35 μm mesh treatment, most likely because the two insects were insufficient or too immature at the third-instar stage for vigorous feeding. We also found that carbon transfer to ponderosa pine shoots and roots increased with declining donor Douglas-fir isotope content, suggesting that increasing severity of defoliation (causing lowered donor isotope uptake) stimulated the belowground flush to networked pine. With manual defoliation, the interior Douglas-fir exported carbon compounds to roots, a behavioral strategy known for helping trees survive subsequent defoliations^39^. The belowground pulses of labile C to roots were then transported to the extramatrical mycorrhizal network, as indicated by the significant C transfer to ponderosa pine receivers. Taken together, the difference in severity between the two defoliation methods and negative relationship between donor and receiver isotope content, supported our expectation that carbon export would increase with defoliation injury. Although our insect treatment was insufficient to elicit a response, we predict that severe sustained insect defoliation would elicit C transfer of similar magnitude as manual defoliation found in this study, but future research is still needed to quantify these effects in the greenhouse and in situ.

Belowground C transfer in the manual defoliation treatment could have occurred via three alternative pathways: soils, mycorrhizal networks, or roots. We excluded the possibility for aboveground communication via volatiles^40^ by covering donors with air-tight plastic bags during defoliation, thus isolating belowground communication pathways^41^. If root exudates transferred via the soil pathway, we would have detected isotope in receivers in both the 0.5 μm mesh and no mesh treatments. If transfer occurred via roots, as shown by Fraser & Lieffers^42^ in *Pinus contorta* Douglas, we would have detected isotope in receivers in the no mesh treatment. Instead, we found no transfer when we excluded mycorrhizal networks with the 0.5 μm mesh, or permitted root-root contact in the no mesh treatment. These results suggest C transfer only occurred through mycorrhizal networks.

As expected, based on previous research by Bingham & Simard^27^, the presence of root competition in the no mesh treatment reduced or masked mycorrhizal C transfer to ponderosa pine. It is possible that any C movement through the intact mycorrhizal network in the no mesh was bi-directional, as shown in Simard et al.^25^ and Philip et al.^26^, but that interior Douglas-fir was a stronger competitor than ponderosa pine for this C pool. The strong competitive ability of interior Douglas-fir roots to acquire belowground C is supported by the reduced germination and growth rates of ponderosa pine in the no mesh versus mesh treatments, where roots of interior Douglas-fir could freely intermingle with those of ponderosa pine. That competitive effects of interior Douglas-fir were driven primarily by belowground rather than aboveground processes is indicated by its smaller shoot stature than ponderosa pine, precluding interior Douglas-fir from pre-empting light from ponderosa pine.

Our data do not support a ‘mesh effect’, where restricted access to grazing invertebrates or greater water retention in the 35 μm mesh bags may have benefitted ponderosa pine growth and hence its sink strength for transferred C. If there were such a mesh effect, we would have found high C transfer rates in the 0.5 μm as well as in the 35 μm mesh bags, but we did not. Nor did we find an effect of mesh presence or mesh size on water available to interior Douglas-fir or ponderosa pine within a pot. Moreover, mesh bags were filled with native soil that likely contained as many invertebrate grazers as soil in the rest of the pot. Therefore, our ability to detect C transfer to ponderosa pine in 35 μm mesh but not in the no mesh treatment was not due to artifacts caused by the presence of the mesh itself, but is most plausibly explained by the absence of root competition from interior Douglas-fir.

Carbon transfer from interior Douglas-fir to ponderosa pine through the mycorrhizal network may have occurred along a carbon or foliar nutrient source-sink gradient^43^. It is possible that C source-sink strength alone played a role in regulating C transfer. Defoliation likely stimulated interior Douglas-fir to rapidly export labile C from enriched roots to the mycorrhizal network^39^, thus increasing the source strength, while the rapid growth rate of ponderosa pine would at the same time have created a large sink strength. In this case, the high amount of C transferred to receiver shoots would have moved via the xylem or transpiration stream as carbohydrates, drawn by high transpiration rates of the ponderosa pine shoots^44^. The preferential movement of labelled C we found to receiver shoots has also been found by others^23^,^25^, including cases where sink strength shifts over growing seasons^8^,^31^,^45^, suggesting that sink strength of ponderosa pine was an important driver of C transfer through the mycorrhizal network. Carbon may have alternatively transferred along a foliar nutrient gradient, where C was translocated with nutrient elements as free amino acids across the Hartig net of donors and receivers. Rapid transfer of these nutrient elements in amino acids would meet an urgent nutritional demand of the fast-growing pine because they are essential for enzyme complexes involved in photosynthesis and protein synthesis.

Approximately half of the total C fixed in the present study was partitioned to belowground pools, suggesting very high potential for belowground C transfer and/or sequestration by these dry forest tree species. Most of the fixed ^13^C remained in donor seedlings, but in the manual defoliation treatment, a large portion (6.8%) was transferred to ponderosa pine. This rate of interspecific transfer through mycorrhizal networks is approximately equivalent to the C costs of reproduction^46^, suggesting the transferred C contributed significantly to receiver biosynthesis. The rate of C transfer we observed was similar to that found through mycorrhizal networks between ectomycorrhizal *Betula payrifera* Marsh and Douglas-fir by Simard et al.^25^ and Philip et al.^26^, and between conspecific *Betula nana* pairs in the Arctic tundra by Deslippe & Simard^31^, but is greater than C transfer between conspecific interior Douglas-fir pairs found in temperate forests by Teste et al.^23^ or Bingham & Simard^27^. These comparisons suggest that strong source-sink gradients resulting from differences in species physiology or harsh environmental conditions drive higher C transfer rates than occur between conspecifics in favorable environments.

### Stress signalling

The significant increase in defense enzyme activities in both interior Douglas-fir donors and ponderosa pine receivers after either manual or insect defoliation of interior Douglas-fir supports our second hypothesis that interspecific communication of stress signals would increase the receiver's defense response. In agreement with our expectations, based on Song et al.^2^,^3^, we found that activity of all three defense enzymes in receivers increased more in the 35 μm mesh treatment than 0.5 μm mesh or no mesh treatments, indicating belowground stress signaling occurred predominantly through mycorrhizal networks. The much smaller increases in enzyme activities in receivers in the 0.5 μm mesh and no mesh treatments, suggest a lesser amount of stress signal transmitted through the soil pathway. Stress signals entering the soil pathway would be subject to the same microbial degradation as C exudates, consistent with our observations. Enzyme activities were always lowest in the non-defoliated controls, indicating that mycorrhization per se did not prime the enzymatic defense response (i.e., mycorrhiza-induced resistance), as discussed by Cameron et al.^47^ Hence, we conclude that mycorrhizal networks transmitted chemical signals that elicited the defensive response of ponderosa pine, supporting recent studies in arbuscular mycorrhizal systems by Song et al.^2^,^3^ and Babikova et al.^4^.

Our expectation that insect defoliation would elicit a greater defense response than manual defoliation because of coevolution between tree and insect species was generally not met. As for C transfer, this was likely due to the low feeding efficacy of western spruce budworms used. However, one exception supported our expectation. The POD activity in insect defoliated donors peaked 24 hours after it did so in the manually defoliated donors, but it then remained higher in the insect defoliated donors. Moreover, despite a lower extent of defoliation by the insect versus manually, the defense response in both donors and receivers were generally similar in magnitude. These results demonstrate that light insect feeding on donors can elicit a strong and rapid defense response in receivers. They support the idea that regulation of plant defense compound production is tightly attuned to herbivore attack, as expected from long standing coevolved relationships between forest trees and herbivores. Our results also show that an additional coevolved interaction, the mycorrhizal symbiosis, and its ability to integrate the plant and fungal community in a network, influences the secondary chemistry of the conifers.

Production of defense enzymes occurred in both donors and receivers 24 h after injury, suggesting stress signals were rapidly exported to the mycorrhizal network. Thus, as defoliated interior Douglas-fir was exporting carbon to ponderosa pine through the mycorrhizal network, it was also transferring defense signals. The healthy ponderosa pine could then induce a defense response to protect itself against a possible attack. Signal transduction is expected to be much faster than the rate of carbon transfer because signal molecules are smaller than carbohydrates or amino acids^48^, enabling them to move more quickly within hyphal networks via cytoplasmic streaming. Although we measured C transfer only once after 6 d, a previous ^14^C autoradiography study showed that it takes at least 3 d for C to transfer from donors to receivers through ectomycorrhizal networks^49^. By contrast, Song et al.^3^ found that the signal molecule jasmonate travelled through the arbuscular mycorhizal network within 6 h of donor insect infestation based on observations of jasmonate accumulation and jasmonate-response gene transcripts in receiver tomato plants. Jasmonate signaling from shoots to roots is well known to play a key role in plant defense response to insect herbivory^50^,^51^. Recent research on electrical signals produced by plants in response to mechanical and insect chewing damage also raises the possibility, however, that the defense response was electrically induced via membrane depolarization events^52^,^53^.

### Host specificity

The communication we observed between interior Douglas-fir and ponderosa pine in response to mechanical and insect defoliation of interior Douglas-fir suggests that the damage elicited a general response. The networking fungus may have acted to protect its net carbon source, by allocating carbon and signals to the healthy, more reliable ponderosa pine. In unstable environments, such as ecosystems under stress and experiencing species turnover as a result of climate change, the mycorrhizal network may therefore benefit from transferring carbon and defense signals interspecifically, favoring hosts that can supply more carbon^54^. It is possible, therefore, that mycorrhizal network-based transfers and signals may evolve to be more generic in stressful environments. The response of ponderosa pine to a stress signal from interior Douglas-fir may have a large cost and little benefit if the damaging agent is host-specific, but be worth investing in constitutive defense enzymes if the damage, as in the manual defoliation treatment, is non-specific. Western spruce budworm is a herbivore of interior Douglas-fir, and to a lesser degree *Larix, Picea* and *Abies* species, so it is intriguing that ponderosa pine mounted a defense response to the attack on its interspecific neighbor. That the defense response occurred in response to host-specific and host-generalist damage suggests that the defense signal itself was a generic signal (e.g., jasmonate). It is also possible that ponderosa pine responds particularly well to abiotic damage and broad herbivore taxa^55^. The decoupling of carbon and defense signal transfer in this study, evident in the differential manual versus insect defoliation effect, suggests that interspecific carbon and defense signal transfer occurred with host-generalist damage (i.e., mechanical damage that can occur in response to abiotic stresses such as wind or drought), but that interspecific signal transduction was possible even with host-specific herbivore damage. Because of these defoliator treatment differences, carbon that was transferred was therefore unlikely a constituent of the defense signal. Further research is needed to understand the compounds, mechanisms, specificity and fitness consequences of communication through mycorrhizal networks.

### Conclusions

We found that mycorrhizal networks transferred physiologically significant levels of photosynthate-derived C and transmitted interspecific stress signals that elicited defense responses in ponderosa pine following manual and insect defoliation of interior Douglas-fir. These results show that mycorrhizal networks are mediators of interactions among trees of different species and defoliators, and therefore likely play a critical role in the defense response and recovery of forests from either abiotic damage or insect outbreaks. The direct pathway of carbon and stress signal transfer through mycorrhizal networks to interspecific plant targets may facilitate shifts in forest composition predicted with climate change.

In many forests of western North America, insect pest epidemics and summer droughts exacerbated by climate change are leaving vast landscapes of dead trees. As these forests regenerate, they are expected to undergo domain shifts to new, hopefully productive stable states of different forest vegetation composition. Our research shows that mycorrhizal networks are positioned to play important roles in facilitating regeneration of migrant species that are better adapted to warmer climates and primed for resistance against insect attacks. These results point to the importance of conservation practices maintaining all of the parts and processes of these highly interconnected forest ecosystems to help them deal with new stresses brought by our changing climate.

## Author Contributions

Y.Y.S., S.W.S. and R.S.Z. designed the research; Y.Y.S. performed the research; Y.Y.S., S.W.S. and R.S.Z. analyzed data; S.W.S. and Y.Y.S. wrote the paper; and A.C. and W.W.M. revised the manuscript.

## Supplementary Material

Supplementary Information SUPPLEMENTARY MATERIAL
