# Supplementary material for: Defoliation of interior Douglas-fir elicits carbon transfer and stress signalling to ponderosa pine neighbors through ectomycorrhizal networks
Source: Sci Rep. 2015 Feb 16;5:8495. doi: 10.1038/srep08495 (PMC4329569; doi:10.1038/srep08495)
Supplement: Supplementary Information — SUPPLEMENTARY MATERIAL [file srep08495-s1.doc]

**SUPPLEMENTARY MATERIAL**

Defoliation of interior Douglas-fir elicits carbon transfer and stress signalling to ponderosa pine neighbors through ectomycorrhizal networks

Yuan Yuan Song^1^, Suzanne W. Simard^2^*, Allan Carroll^2^, William W. Mohn^3^, and Ren Sen Zeng^1^

^1^College of Life Sciences, Fujian Agriculture and Forestry University, Jinshan, Fuzhou 350002, P.R. China

^2^Department of Forest and Conservation Sciences, University of British Columbia, Vancouver, British Columbia, V6T 1Z4, Canada

^3^Department of Microbiology & Immunology, Life Sciences Institute, University of British Columbia, Vancouver, BC, V6T 1Z3, Canada

*Correspondence to: [suzanne.simard@ubc.ca](mailto:suzanne.simard@ubc.ca), Department of Forest and Conservation Sciences, University of British Columbia, Vancouver, British Columbia, Canada, V6T 1Z4, phone: 604-822-1955, fax: 604-822-9102

**SUPPLEMENTARY MATERIAL**

Figure S1. Soil volumetric water content of interior Douglas-fir donors and ponderosa pine seedlings in the transfer pathway treatments. Means with different letters for each pathway differed significantly at α=0.05.

Figure S2. Levels of enzyme activity (POD, PPO, SOD) in needles of donor interior Douglas-fir

following different defoliation and transfer pathway treatments.

Figure S3. Relationships between donor and receiver enzyme activity (U -1 g-1 FW) for each enzyme (POD, PPO and SOD) and time step (0, 24, 48 and 72 h) following defoliation.

(a)

(b)

Figure S4. Relationships between receiver excess ^12^C-equivalent (mg) and receiver enzyme activity in needles and roots for (a) PPO and (b) SOD at the 48 and 72 h time steps following defoliation.
